# CD26 expression is attenuated by TGF‐β and SDF‐1 autocrine signaling on stromal myofibroblasts in human breast cancers

**DOI:** 10.1002/cam4.2249

**Published:** 2019-05-29

**Authors:** Yoshihiro Mezawa, Yataro Daigo, Atsushi Takano, Yohei Miyagi, Tomoyuki Yokose, Toshinari Yamashita, Chikao Morimoto, Okio Hino, Akira Orimo

**Affiliations:** ^1^ Department of Molecular Pathogenesis, Graduate School of Medicine Juntendo University Tokyo Japan; ^2^ Center for Antibody and Vaccine Therapy, Institute of Medical Science Research Hospital The University of Tokyo Tokyo Japan; ^3^ Department of Medical Oncology and Cancer Center Shiga University of Medical Science Otsu Japan; ^4^ Molecular Pathology and Genetics Division Kanagawa Cancer Center Research Institute Yokohama Japan; ^5^ Department of Pathology Kanagawa Cancer Center Yokohama Japan; ^6^ Department of Breast and Endocrine Surgery Kanagawa Cancer Center Yokohama Japan; ^7^ Department of Therapy Development and Innovation for Immune Disorders and Cancers Juntendo University Tokyo Japan

**Keywords:** breast cancer, dipeptidyl peptidase 4, myofibroblasts, stromal cell‐derived factor 1, TGF‐beta

## Abstract

Human breast carcinoma‐associated fibroblasts (CAFs) increasingly acquire both transforming growth factor‐β (TGF‐β) and stromal cell‐derived factor‐1 (SDF‐1) signaling in an autocrine fashion during tumor progression. Such signaling mediates activated myofibroblastic and tumor‐promoting properties in these fibroblasts. CD26/dipeptidyl peptidase‐4 is a serine protease that cleaves various chemokines including SDF‐1. Stromal CD26 expression is reportedly undetectable in human skin squamous cell carcinomas. However, whether stromal CD26 expression is also downregulated in human breast cancers and which stromal cells potentially lack CD26 expression remain elusive. To answer these questions, sections prepared from 239 human breast carcinomas were stained with antibodies against CD26 and α‐smooth muscle actin (α‐SMA), a marker for activated myofibroblasts. We found that tumor‐associated stroma involving α‐SMA‐positive myofibroblasts stained negative or negligible for CD26 in 118 out of 193 (61.1%) tumors, whereas noncancerous stromal regions of the breast showed considerable staining for CD26. This decreased stromal CD26 staining in tumors also tends to be associated with poor outcomes for breast cancer patients. Moreover, we demonstrated that CD26 staining is attenuated on stromal myofibroblasts in human breast cancers. Consistently, CD26 expression is significantly downregulated in cultured CAF myofibroblasts extracted from human breast carcinomas as compared to control human mammary fibroblasts. Inhibition of TGF‐β or SDF‐1 signaling in CAFs by shRNA clearly upregulated the CD26 expression. Taken together, these findings indicate that CD26 expression is attenuated by TGF‐β‐ and SDF‐1‐autocrine signaling on stromal myofibroblasts in human mammary carcinomas, and that decreased stromal CD26 expression has potential as a prognostic marker.

## 1 INTRODUCTION

Desmoplastic stroma rich in α‐smooth muscle actin (α‐SMA)‐positive myofibroblasts, a hallmark of activated fibroblasts, is frequently observed in various human carcinomas including those of the breast, prostate, pancreas, lung, and colon.^1^, ^2^, ^3^ In contrast, such myofibroblasts are rarely identified within nontumor stromal regions. Large numbers of myofibroblasts and α‐SMA‐negative fibroblasts often comprise carcinoma‐associated fibroblasts (CAFs) in the tumor‐associated stroma.

CAFs rich in myofibroblasts produce multiple growth factors, cytokines, chemokines, and exosomes which influence a wide variety of tumor hallmarks.^4^, ^5^, ^6^, ^7^ We and others have previously described that CAF‐secreted transforming growth factor‐β (TGF‐β) and stromal cell‐derived factor‐1 (SDF‐1) promote the growth of apposed carcinoma cells in a paracrine fashion.^8^, ^9^, ^10^ These stromal cytokines also allow establishment of cross‐communicating TGF‐β and SDF‐1 autocrine signaling by acting on their cognate receptors, resulting in the induction and maintenance of activated, tumor‐promoting properties of CAFs without ongoing interaction with tumor cells during tumor progression.^8^, ^9^, ^10^

Possible CAF markers including α‐SMA, fibroblast activation protein alpha, fibroblast‐specific protein‐1 (also known as S100A4), tenascin‐C, platelet‐derived growth factor receptor‐α/β, and podoplanin have been identified.^6^, ^11^ Although these CAF markers are useful for predicting the outcomes of some human breast carcinoma cohorts,^12^, ^13^ none fully or exclusively identifies activated tumor‐promoting CAFs due to various differences in fibroblast populations, as exemplified by resident fibroblasts and bone‐marrow‐derived progenitors present in tumors. Therefore, no conventional stromal marker has yet been identified for use in routine prognostic determinations for human carcinomas including those of the breast.

CD26**/**dipeptidyl peptidase‐4 (DPP‐4) is expressed by a wide variety of cell types and is involved in T‐cell activation, immune regulation, cell adhesion, signal transduction, apoptosis, and so on.^14^, ^15^, ^16^ Both membrane‐bound and soluble forms of CD26 have serine protease activity that preferentially cleaves dipeptides from the N‐terminal region of peptides and proteins with a proline or alanine residue in the penultimate position.^14^, ^15^ Stromal CD26 expression has been shown to be remarkably attenuated in human skin and oral squamous cell carcinomas (SCCs).^17^, ^18^ However, whether stromal CD26 expression is commonly downregulated in different cancer types remains unclear. Moreover, the stromal cell types potentially lacking CD26 expression, as well as the molecular mechanisms underlying attenuated stromal CD26 expression, has not as yet been elucidated.

In the present study, we demonstrated that CD26 expression is attenuated via TGF‐β and SDF‐1 autocrine signaling on stromal myofibroblasts in human breast carcinomas. This downregulated stromal CD26 expression in tumors is associated with poor outcomes for breast cancer patients.

## 2 MATERIALS AND METHODS

### 2.1 Cell culture

Human mammary fibroblasts were extracted from a healthy breast tissue specimen that had been obtained by reduction mammoplasty prior to primary culture and immortalization with human telomerase reverse transcriptase as described previously.^9^ Human breast exp‐CAF2 cells and the corresponding control human mammary fibroblasts were also employed.^9^ These cells were cultured in DMEM high glucose GlutaMAX™ (Gibco) supplemented with 10% fetal bovine serum (FBS) and 1% Pen Strep (100 U/mL penicillin and 100 μg/mL streptomycin) (Gibco). MCF10DCIS.com (DCIS) cells were purchased from Asterand Bioscience. MDA‐MB‐231 cells were purchased from American Type Culture Collection. These breast cancer cells were cultured in DMEM/F‐12, GlutaMAX™ (Gibco) supplemented with 1% PenStrep (Gibco) with 5% FBS (DCIS cells) or 10% FBS (MDA‐MB‐231 cells).

### 2.2 Immunohistochemistry

The use of formalin‐fixed paraffin‐embedded (FFPE) tissue specimens of breast cancer in this study was approved by the Juntendo University ethics review board. FFPE invasive breast carcinomas were prepared from breast cancer patients who had received either preoperative chemotherapy or hormone therapy. Three‐micrometre thick sections were prepared and deparaffinized. The slides were then treated with 0.3% H_2_O_2_ in methanol for 20 minutes at room temperature. Antigen retrieval was performed by autoclaving in citrate buffer at pH 6.0 for 20 minutes at 121°C. The slides were incubated with primary antibody at 4°C overnight. Secondary antibody was incubated for 1 hour at room temperature. 3,3′‐diaminobenzidine was used as the chromogen followed by hematoxylin counterstaining.

Semiquantification of CD26‐positive fibroblasts was performed as follows. Ten different fields on both cancerous and noncancerous regions rich in stroma of the breast were captured per slide using ×400 magnification under a microscope. The stromal cells exhibiting a typical fibroblast‐like spindle‐shape were regarded as “fibroblast‐like cells.” Tumor cells, vascular endothelial cells, white blood cells, and adipocytes were also discriminated morphologically. CD26‐positive fibroblast‐like cells (%) were calculated as the ratio of the number of CD26‐positive fibroblast‐like cells relative to that of all fibroblast‐like cells in cancerous and noncancerous regions of specimens obtained from 10 breast cancer patients.

### 2.3 Tissue microarray

Tissue microarrays were constructed using 239 formalin‐fixed primary breast cancer specimens, as reported previously.^19^ Paraffin‐embedded tissue sections were obtained from specimens that had been surgically resected at Kanagawa Cancer Center. Individual institutional ethics committees approved this study and the use of all clinical materials. Experiments were performed in accordance with all guidelines and regulations indicated by these committees. The tissue area for sampling was selected based on visual alignment with the corresponding hematoxylin and eosin–stained section on a slide. Several tissue cores (diameter 0.6 mm; height 3‐4 mm) taken from a donor tumor block were placed into a recipient paraffin block using a tissue microarrayer (Beecher Instruments). Resulting microarray blocks were used for immunohistochemical analysis. The sections were stained using anti‐CD26 and ‐α‐SMA antibody according to the conditions described in the immunohistochemistry section. Immunohistochemical scores for CD26 and α‐SMA expressions in stromal fibroblast‐like cells were determined by a researcher with no prior knowledge of the clinicopathological results, as follows: negative and negligible (<10% of total area) and moderately and significantly positive (more than 10% of total area) for CD26 staining, and weakly positive (<50% of total area) and strongly positive (more than 50% of total area) for α‐SMA staining.

### 2.4 Statistical analysis

*P* < 0.05 was considered to indicate a statistically significant difference, as indicated by * in graphs. When the *P*‐value was < 0.001, it was indicated as **. To investigate the associations of stromal CD26 expression with patient characteristics in stromal α‐SMA‐positive breast cancer, Fisher's exact test was performed.

## 3 RESULTS

### 3.1 Attenuated CD26 expression on stromal myofibroblasts in human breast carcinomas

**Figure 1:**
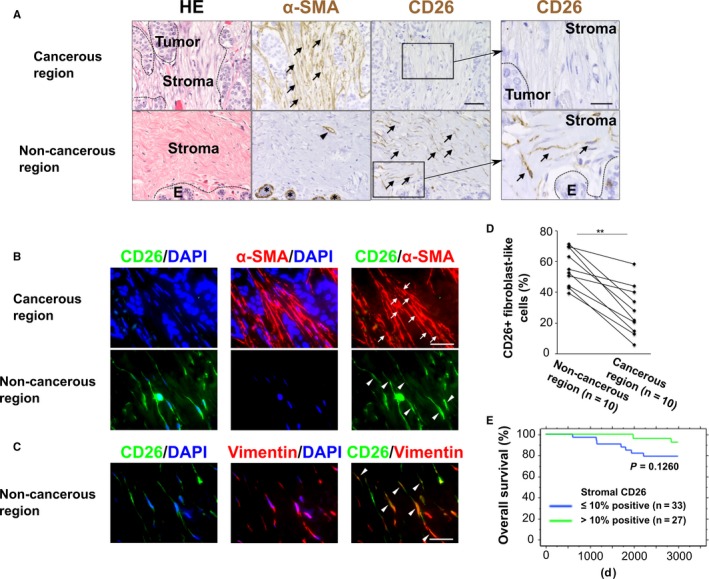
Attenuated CD26 staining on stromal myofibroblasts in human breast cancers. A, Hematoxylin and eosin (HE) staining and immunohistochemistry of sections prepared from human breast cancer tissue specimens using anti‐CD26 or ‐α‐smooth muscle actin (α‐SMA) antibodies. CD26^+^ fibroblast‐like cells in a noncancerous region and α‐SMA^+^ myofibroblasts in a cancerous region are indicated by arrows. α‐SMA^+^ pericytes associated with a blood vessel (arrowhead) and α‐SMA^+^ myoepithelial cells surrounding normal mammary glands (asterisks) are also shown. Scale bar, 50 µm. Right column, magnified images of CD26 staining. Scale bar, 20 µm. B, Double immunofluorescence of sections prepared from the human breast tissue specimens. CD26^−^ α‐SMA^+ ^myofibroblasts (arrows) in a cancerous region and CD26^+^ α‐SMA^−^ fibroblast‐like cells (arrowheads) in a noncancerous region are shown. Scale bar, 30 µm. C, Double immunofluorescence of sections prepared from the noncancerous region of the human breast cancer tissue. CD26^+^ vimentin^+^fibroblasts are indicated by arrowheads. Scale bar, 30 µm. D, Quantification of CD26‐positive fibroblast‐like cell populations in tumor‐associated stroma of 10 human breast cancer patients. Human breast tissues including noncancerous and cancerous regions were stained with anti‐CD26 antibody. **P < 0.001 by paired *t*‐test. E, Kaplan‐Meier plot indicating overall survival of breast cancer patients. Patients were grouped according to the indicated CD26 staining in tumor‐associated stroma rich in myofibroblasts (>50% positive for α‐SMA staining). The *P*‐value was determined based on the Log‐rank test. Abbreviation: E, normal human mammary epithelium

To examine whether stromal CD26 expression is attenuated in human breast carcinomas, paraffin sections were prepared from human breast cancer specimens and stained with anti‐CD26 or ‐α‐SMA antibody. Few CD26‐positive cells were detected in tumor stroma including an abundance of α‐SMA‐positive myofibroblasts, while a larger number of CD26‐positive fibroblast‐like cells were present in a noncancerous region lacking myofibroblasts of the breast far from the outer tumor margin (Figure 1A). A subset of lymphocytes also stained positive for CD26 (Figure S1A), while vascular endothelial cells were negative for CD26 (Figure S1B). Moreover, most breast cancer cells stained negative for CD26 (Figure 1A), consistent with previous reports.^20^, ^21^

To address whether CD26 expression is attenuated on stromal myofibroblasts in human breast tumors, double immunofluorescence was performed using both anti‐CD26 and ‐α‐SMA antibodies. CD26 expression was barely detected on α‐SMA‐positive myofibroblasts in tumor‐associated stroma (Figure 1B). In sharp contrast, considerable CD26 expression was observed in α‐SMA‐negative stromal fibroblasts expressing vimentin, a marker of the mesenchymal cell type in noncancerous regions (Figure 1B,C).

To extend this observation, we performed immunohistochemistry on specimens from 10 patients in total (Table 1). Of note, in all of the examined breast cancer patients, CD26‐positive fibroblast‐like cell proportions were significantly decreased in the tumor‐associated stroma as compared to those in the corresponding noncancerous stroma of the same breast (Figure 1D). Furthermore, we investigated another patient cohort including 239 breast cancers by immunohistochemistry using anti‐CD26 and ‐α‐SMA antibodies. Stromal myofibroblasts were stained positive for α‐SMA in 193 out of 239 (80.8%) tumors. Stromal CD26 staining was also negative or negligible (<10% positive) in 118 out of the 193 (61.1%) tumors including stromal myofibroblasts, and showed no significant correlations with any pathological parameters (Table 2). This decreased stromal CD26 staining tended to be associated with poorer outcomes for breast cancer patients with tumors rich in stromal myofibroblasts than did moderately and significantly positive stromal CD26 staining (Figure 1E). Collectively, these data indicate that the attenuated CD26 expression on stromal myofibroblasts in tumors may contribute to poor outcomes in breast cancer patients.

**Table 1:** Patients' information

Case	Sex	Age	Histology	Histological grade	ER	PR	HER2	Stage
1	F	40	IDC and DCIS	2	90%	90%	2+	IIB
2	F	47	IDC and DCIS	2	−	−	1+	IA
3	F	75	IDC and DCIS	3	−	−	3+	IIB
4	F	61	IDC and DCIS	2	−	−	1+	IIB
5	F	55	IDC and DCIS	2	>90%	>70%	2+	IA
6	F	44	IDC	2	>90%	>80%	2+	IA
7	F	72	IDC and DCIS	3	−	−	1+	IIA
8	F	53	IDC and DCIS	1	>90%	>90%	2+	IA
9	F	76	IDC and DCIS	3	−	−	1+	IA
10	F	39	IDC and DCIS	2	>90%	>90%	2+	IIB

Note

Information about patients whose breast tumor‐derived FFPE tissue was used in this study for immunohistochemistory: diagnosis was performed by pathologists in the Juntendo University Hospital.^22^, ^23^, ^24^ ER and PR positive cell number (%) and immunoreactivity of HER2 were determined according to ASCO guidelines.^23^, ^24^ Stage was determined according to UICC TNM classification.

Abbreviations: −, negative; DCIS, ducutal carcinoma in situ; ER, estrogen receptor; F, female; FFPE, formalin‐fixed paraffin‐embedded; HER2, human epithelial growth factor receptor type 2; IDC, invasive ductal carcinoma; PR, progesterone receptor.

**Table 2:** Associations of CD26 expression in tumor stroma containing myofibroblasts with clinical parameters of 193 breast cancer patients

Parameters		Total n = 193	Stromal CD26 negative and negligible staining (up to 10%) n = 118	Stromal CD26 moderate and significant staining (more than 10%) n = 75	*P*‐value
Age (years)	~65	148	96	52	0.0576
	66~	45	22	23	
Grading	0	25	15	10	0.7677 (grading 0 and 1 vs 2 and 3)
	1	66	42	24	
	2	53	35	18	
	3	49	26	23	
pT factor	T1	74	44	30	0.762
	T2‐3	119	74	45	
pN factor	N0	102	58	44	0.237
	N1‐2	91	60	31	
ER	Positive	135	89	46	0.0527
	Negative	58	29	29	
HER2	Positive	28	16	12	0.678
	Negative	165	102	63	

Abbreviations: ER, estrogen receptor; HER2, human epithelial growth factor receptor type 2.

### 3.2 Decreased CD26 expression on tumor‐promoting human breast CAFs

**Figure 2:**
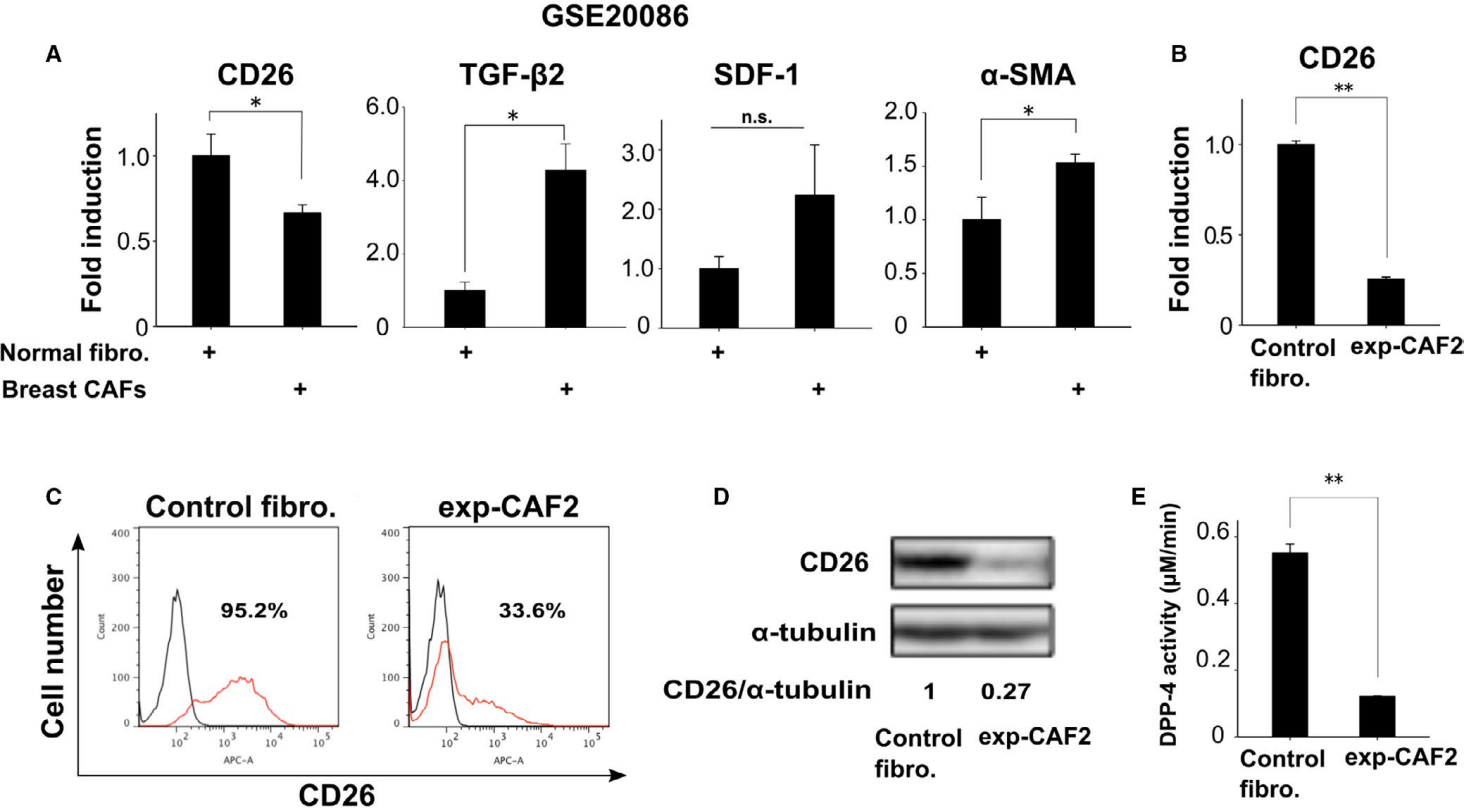
Downregulated CD26 expression on human breast carcinoma‐associated fibroblasts (CAFs). A, mRNA expressions of the indicated genes in normal human mammary fibroblasts and CAFs (n = 6) using public microarray data in GSE20086. B, Real‐time PCR of control human mammary fibroblasts and exp‐CAF2 cells for CD26 expression. C, Flow cytometry of the indicated cells using anti‐CD26 antibody (red line) or the control IgG (black line). The number of CD26‐positive cell populations (%) is also indicated. D, Western blotting of the indicated cells using anti‐CD26 antibody. The ratio of the signal intensity of CD26 relative to α‐tubulin is indicated. E, Dipeptidyl peptidase‐4 (DPP‐4) activity in whole cell lysates derived from the indicated cells (n = 3). **P < 0.001 and *P < 0.05 by Student's *t*‐test. Error bars, SE. Abbreviation: n.s., not significant

As CD26 expression was attenuated on stromal myofibroblasts in human breast carcinomas, we investigated whether CD26 expression is also downregulated in primary cultured CAFs extracted from human breast carcinomas, compared to the corresponding control fibroblasts isolated from the adjacent noncancerous tissues in same patients using public gene expression data.^25^ A significantly lower level of CD26 mRNA expression was detected in myofibroblastic CAFs that presumably acquired TGF‐β and SDF‐1 autocrine signaling, as exemplified by increased TGF‐β2 and SDF‐1 mRNA expression^9^, ^26^ (Figure 2A).

We also employed exp‐CAF2 cells raised from immortalized human mammary fibroblasts that had been incubated with MCF‐7*‐ras* breast cancer cells in the tumor xenograft and then extracted from the developing tumor for subsequent expansion in culture.^27^ As mentioned above, the exp‐CAF2 cells increasingly acquired myofibroblastic and tumor‐promoting traits via establishment of TGF‐β and SDF‐1 autocrine signaling through interaction with carcinoma cells during tumor progression.^9^ We indeed found CD26 mRNA expression to be downregulated in exp‐CAF2 cells, by 74.4% as compared to the control human mammary fibroblasts that were minimally activated, in terms of myofibroblastic and tumor‐promoting properties (Figure 2B). Moreover, cell surface CD26 expression was decreased on exp‐CAF2 cells by 64.7%, as demonstrated by flow cytometry (Figure 2C). In addition, CD26 protein expression and DPP‐4 activity (CD26 peptidase activity) were decreased in exp‐CAF2 cells by 73.0% and 78.2%, respectively (Figure 2D,E). Taken together, these findings indicate that CD26 expression and DPP‐4 activity are significantly attenuated on myofibroblastic CAFs with activated TGF‐β and SDF‐1 autocrine signaling.

### 3.3 CD26 expression attenuated by TGF‐β‐Smad2/3 autocrine signaling on CAFs

We next investigated how CD26 expression is downregulated on CAFs. Given the increasingly activated TGF‐β‐ and SDF‐1‐autocrine signaling in exp‐CAFs during tumor progression,^9^ we reasoned that such signaling might contribute to attenuation of CD26 expression on these cells.

**Figure 3:**
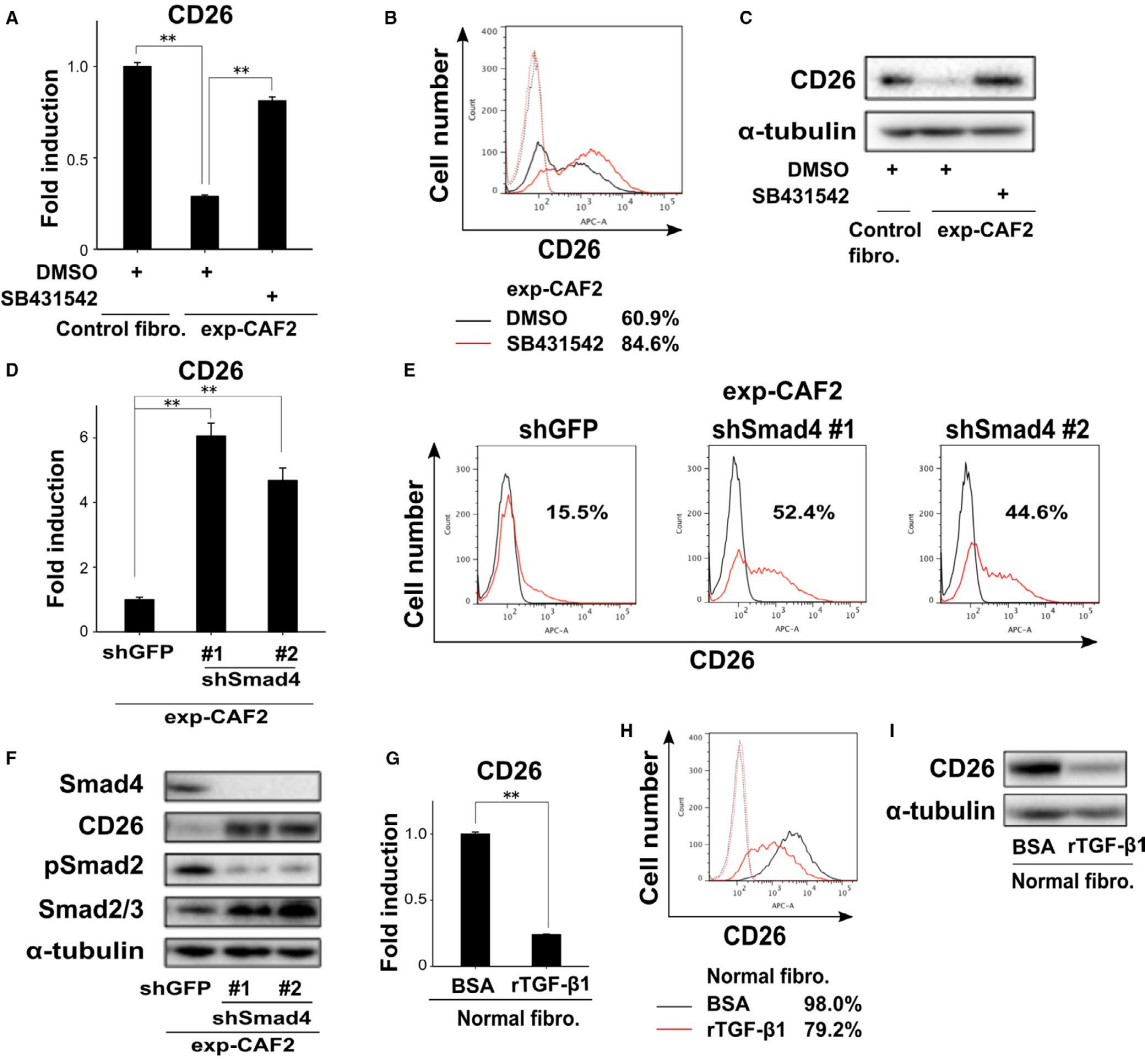
Decreased CD26 expression mediated by transforming growth factor‐β (TGF‐β)‐Smad2/3 autocrine signaling on carcinoma‐associated fibroblasts (CAFs). A, Real‐time PCR of the indicated fibroblasts treated with dimethyl sulfoxide (DMSO) or SB431542 for 24 h to measure CD26 expression. B, Flow cytometry of exp‐CAF2 cells treated with DMSO (black line) or SB431542 (red line) for 48 h using anti‐CD26 antibody (solid line) or the control IgG (dotted line). The number of CD26‐positive cell populations (%) is shown. C, Western blotting of the described cells treated with DMSO or SB431542 for 48 h. D, Real‐time PCR of exp‐CAF2 cells expressing GFP‐ and Smad4‐shRNA (#1 and #2) for CD26 expression. E, Flow cytometry of indicated cells using anti‐CD26 antibody (red line) or the control IgG (black line). The number of CD26‐positive cell populations (%) is depicted. F, Western blotting of exp‐CAF2 cells expressing GFP‐ and Smad4‐shRNA (#1 and #2). G, Real‐time PCR of human mammary fibroblasts treated with bovine serum albumin (BSA) or recombinant TGF‐β1 (10 ng/mL) for 24 h to measure CD26 expression. H, Flow cytometry of human mammary fibroblasts treated with BSA (black line) or TGF‐β1 (10 ng/mL, red line) for 48 h using anti‐CD26 antibody (solid line) or the control IgG (dotted line). The number of CD26‐positive cell populations (%) is depicted. I, Western blotting of human mammary fibroblasts treated with BSA or recombinant TGF‐β1 (10 ng/mL) for 48 h **P < 0.001 by Student's *t*‐test. Error bars, SE

To examine this possibility, exp‐CAF2 cells were treated with SB431542, an inhibitor for TGF‐β receptor I kinase activity, which is crucial for phosphorylation of the downstream proteins represented by Smad2/3.^28^ CD26 expression was significantly upregulated at both the mRNA and protein levels on the resulting exp‐CAF2 cells relative to the effect of the control dimethyl sulfoxide treatment (Figure 3A‐C).

We also sought to the determine roles of the canonical TGF‐β‐Smad2/3 pathway in the attenuated CD26 expression on CAFs. To this end, we generated two different shRNA constructs against Smad4, which is a central mediator of the Smad2/3 signaling to inhibit Smad4 expression in exp‐CAF2 cells. Inhibition of Smad4 expression by shRNA upregulated CD26 mRNA and protein expressions significantly more than did the GFP‐shRNA (Figure 3D‐F). In sharp contrast, the expression level of phosphorylated Smad2 (pSmad2), indicative of the activation of TGF‐β signaling,^28^ was strongly attenuated in exp‐CAF2 cells expressing Smad4‐shRNA (Figure 3F). These data therefore indicate that the TGF‐β‐Smad2/3 signaling pathway is required for maintenance of the attenuated CD26 expression on CAFs.

Given the TGF‐β‐Smad2/3 signaling requirement for the attenuated CD26 expression on CAFs, we also investigated whether this signaling suffices to induce downregulation of CD26 expression. To examine this possibility, human mammary fibroblasts were treated with TGF‐β1. Expression levels of CD26 mRNA and protein were significantly attenuated in these cells (Figure 3G‐I). Taken together, these findings demonstrate that activation of TGF‐β‐Smad2/3 signaling induces and maintains the attenuated CD26 expression on exp‐CAF2 cells.

### 3.4 SDF‐1 signaling and CD26 expression on CAFs

Since SDF‐1 signaling is critical for mediating the myofibroblastic tumor‐promoting trait in CAFs,^9^ we investigated whether this signaling regulates CD26 expression on these cells.

**Figure 4:**
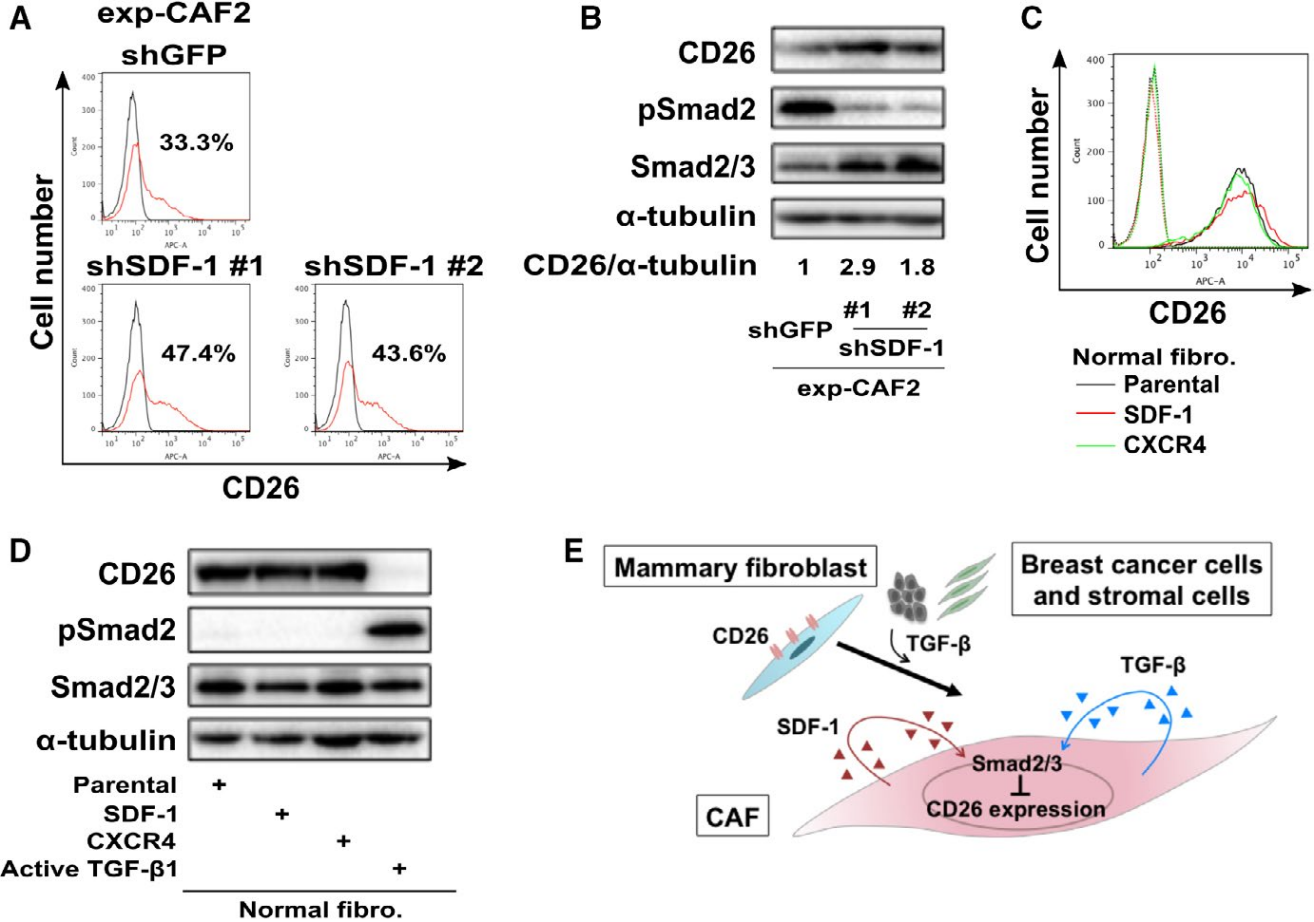
Stromal cell‐derived factor‐1 (SDF‐1) autocrine signaling required for the attenuated CD26 expression on exp‐carcinoma‐associated fibroblasts (CAFs) cells. A, Flow cytometry of indicated cells using anti‐CD26 antibody (red line) or the control IgG (black line). The number of CD26‐positive cell populations (%) is also depicted. B, Western blotting of exp‐CAF2 cells expressing the indicated shRNA. The ratio of the signal intensity of CD26 relative to α‐tubulin is indicated. C, Flow cytometry of human normal mammary fibroblasts (parental, black line) expressing SDF‐1 (red line) or CXCR4 (green line) cDNA construct using anti‐CD26 antibody (solid line) or the control IgG (dotted line). D, Western blotting of human normal mammary fibroblasts expressing the indicated cDNA construct. E, Schematic representation of the attenuated CD26 expression via transforming growth factor‐β (TGF‐β)‐Smad2/3 and SDF‐1 autocrine signaling on human breast CAF myofibroblasts. TGF‐β released from tumor cells and stromal cells attenuates CD26 expression on human mammary fibroblasts. Acquisition of TGF‐β and SDF‐1 autocrine signaling pathways then contributes to maintenance of the attenuated CD26 expression on CAFs during tumor progression

To assess this possibility, CD26 expression was measured on exp‐CAF2 cells expressing SDF‐1‐shRNAs, both of which significantly inhibited SDF‐1 expression (Figure S2A).^9^ Inhibition of SDF‐1 expression upregulated levels of CD26 protein expression on these cells as compared to the effect of GFP‐shRNA, as demonstrated by flow cytometry (Figure 4A) and Western blotting (Figure 4B). Furthermore, pSmad2 expression was also attenuated in exp‐CAF2 cells expressing each of these SDF‐1‐shRNAs (Figure 4B). These findings therefore indicate that SDF‐1 expression is required for the attenuation of CD26 expression via activation of Smad2/3 signaling on exp‐CAF2 cells. This observation is consistent with our previous findings, indicating that SDF‐1 signaling mediates TGF‐β‐Smad2/3 signaling in CAFs.^9^

We next examined whether SDF‐1‐CXCR4 signaling suffices to attenuate the CD26 expression on mammary fibroblasts. To answer this question, a retroviral construct encoding either human SDF‐1 or CXCR4 cDNA was introduced into human mammary fibroblasts (Figure S2B).^9^, ^10^ The levels of CD26 protein expression were similar in parental human mammary fibroblasts and those overexpressing SDF‐1 or CXCR4, as demonstrated by flow cytometry (Figure 4C) and Western blotting (Figure 4D). pSmad2 expressions also differed minimally among all of these fibroblasts (Figure 4D). Conversely, CD26 expression was robustly inhibited on human mammary fibroblasts expressing an active TGF‐β1 cDNA^9^ via increased pSmad2 expression (Figures 4D and S2B), confirming earlier data showing attenuated CD26 expression on the TGF‐β1‐treated mammary fibroblasts (Figure 3I).

Collectively, these findings indicate that SDF‐1 autocrine signaling is required for maintenance of the attenuated CD26 expression on CAFs presumably via Smad2/3 signaling, but is not sufficient for inducing the downregulation of CD26 expression on these cells (Figure 4E). As mentioned above, the activation of TGF‐β‐Smad2/3 signaling was found to both induce and maintain the downregulated CD26 expression on CAFs (Figure 4E).

### 3.5 Roles of decreased DPP‐4 activity on CAFs in TGF‐β and SDF‐1 autocrine signaling

**Figure 5:**
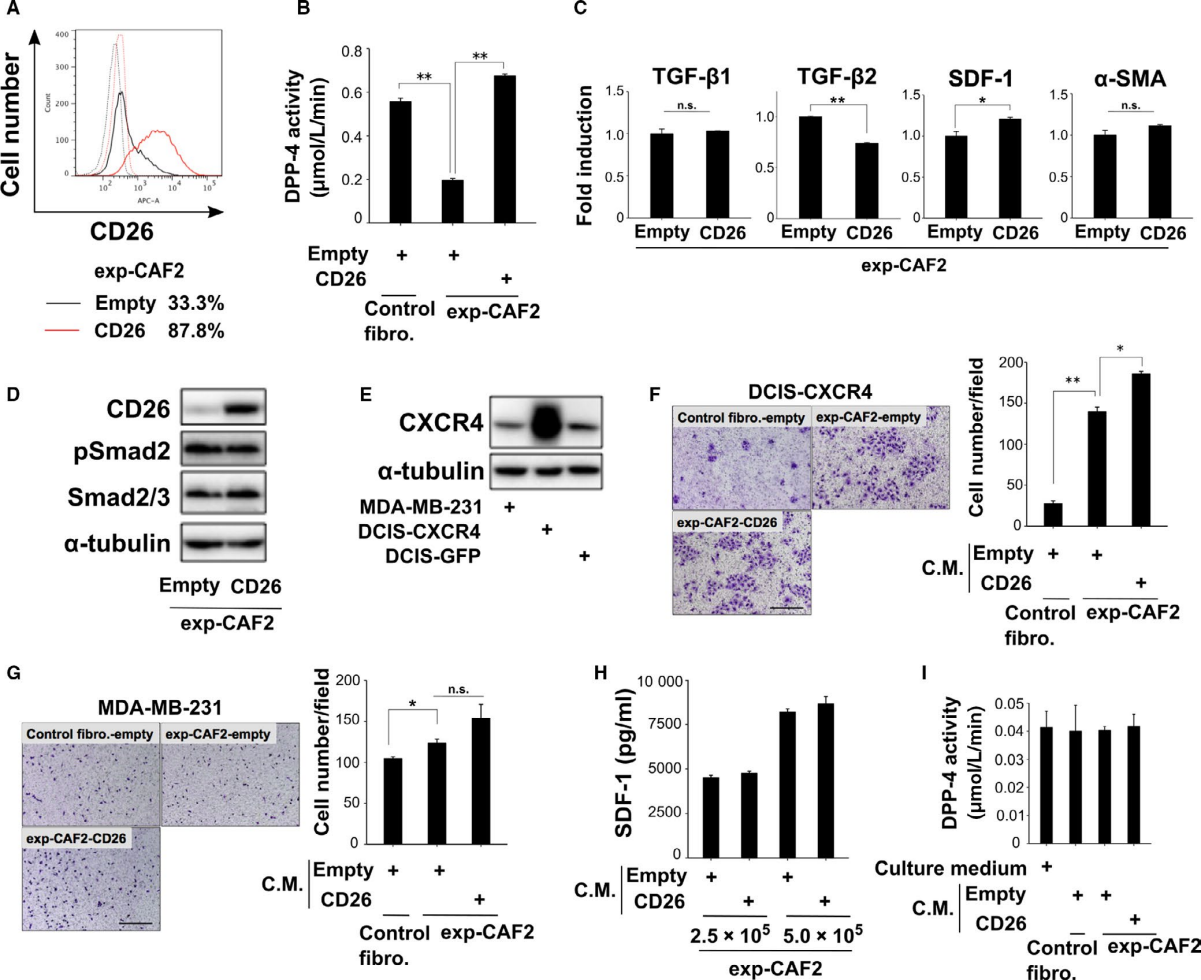
Attenuated CD26 expression is not essential for stromal cell‐derived factor‐1 (SDF‐1)‐autocrine and ‐paracrine signaling in carcinoma‐associated fibroblasts (CAFs). A, Flow cytometry of exp‐CAF2‐empty (black line) or ‐CD26 (red line) cells using anti‐CD26 antibody (solid line) or the control IgG (dotted line). The number of CD26‐positive cell populations (%) is shown. B, Dipeptidyl peptidase‐4 (DPP‐4) activity in the indicated fibroblasts (n = 3). C, Real‐time PCR of indicated cells for transforming growth factor‐β1 (TGF‐β1), TGF‐β2, SDF‐1 and α‐SMA expressions. D,E, Western blotting of the indicated cells. F,G, Boyden chamber cell migration assay of DCIS‐CXCR4 cells (F) or MDA‐MB‐231 cells (G) using conditioned medium (CM) taken from the indicated cells. The cells which had migrated were stained with May‐Grünwald Giemsa at 60 h (DCIS‐CXCR4, F) or at 12 h (MDA‐MB231, G) after the cell seeding. Scale bar, 300 µm. The number of tumor cells which migrated per field is shown (n = 3). H, ELISA of CM taken from the indicated fibroblasts (2.5 × 10^5^ or 5 × 10^5^ cells) for measuring SDF‐1. I, DPP‐4 activity in CM derived from the indicated fibroblasts (n = 3). **P < 0.001 and *P < 0.05 by Student's *t*‐test. Error bars, SE. Abbreviation: n.s., not significant

SDF‐1 is a major substrate for CD26/DPP‐4 peptidase.^14^, ^29^ The resulting failure of transduction of the downstream signaling of CXCR4, an SDF‐1 receptor present on the target cells, attenuates hematopoietic stem/progenitor cell homing,^30^ HIV infection,^31^, ^32^ and cancer cell invasion.^33^, ^34^, ^35^, ^36^ Given these observations, we speculated that the attenuated stromal CD26 expression may influence SDF‐1 autocrine signaling and the myofibroblastic state in CAFs. To this end, a retroviral vector encoding the human CD26 cDNA or the corresponding control empty vector was introduced into exp‐CAF2 cells or control fibroblasts. CD26 protein expression and DPP‐4 activity were markedly increased on exp‐CAF2 cells expressing CD26 (exp‐CAF2‐CD26) as compared to the control vector (exp‐CAF2‐empty) (Figure 5A,B). However, TGF‐β and SDF‐1 autocrine signaling as well as the myofibroblastic trait, as exemplified by TGF‐β1, TGF‐β2, pSmad2, SDF‐1, and α‐SMA expressions were similar in these cells (Figure 5C,D). These data therefore indicate that the attenuated CD26 expression does not significantly contribute to activation of TGF‐β and SDF‐1 autocrine signaling or to the myofibroblastic trait in CAFs.

### 3.6 Roles of attenuated DPP‐4 activity on CAFs in their SDF‐1 paracrine signaling toward apposed carcinoma cells

We have previously demonstrated that CAF‐produced SDF‐1 promotes the growth of nearby breast tumor cells in a paracrine fashion via acting through CXCR4 on these cells.^10^ We thus speculated that decreased levels of membrane and soluble CD26 expressions on CAFs may promote paracrine SDF‐1 signaling toward nearby carcinoma cells.

To investigate this possibility, the biological activity of stromal SDF‐1 was evaluated employing the Boyden chamber cell migration assay using human breast cancer MDA‐MB‐231 cells and DCIS cells overexpressing CXCR4 (DCIS‐CXCR4) (Figure 5E). We observed more robust migration of these cancer cells to be induced by the SDF‐1‐rich medium conditioned by exp‐CAF2‐empty relative to control fibroblast‐empty (Figure 5F,G). Medium derived from exp‐CAF2‐CD26 or exp‐CAF2‐empty cells, when applied to these cancer cells, showed similar tumor cell migration (Figure 5F,G). Furthermore, SDF‐1 protein concentrations were similar in media conditioned by these cells (Figure 5H). Moreover, DPP‐4 activity was nearly undetectable in media obtained from exp‐CAF2‐CD26 and control human mammary fibroblasts abundantly expressing CD26 (Figure 5I), while a markedly higher level of DPP‐4 activity was detected in the whole cell lysate extracted from these fibroblasts (Figure 5B). Collectively, these observations indicate that membrane CD26 is barely shed on exp‐CAF2‐CD26 and human mammary fibroblasts, suggesting that increased membrane CD26 expression by itself may not be enough to inhibit the SDF‐1 activity. However, whether attenuated soluble CD26 production promotes paracrine SDF‐1 signaling from CAFs could not be resolved by the experiments above.

## 4 DISCUSSION

### 4.1 Attenuated CD26 expression in myofibroblastic CAFs is associated with poor outcomes in breast cancer patients

Stromal CD26 expression is known to be barely detectable in different human SCCs.^17^, ^18^ However, whether stromal CD26 expression is also downregulated in human breast carcinomas remains controversial; stromal CD26 expression was reportedly undetectable in a tumor taken from one breast cancer patient,^20^ while its expression was detected in another human breast carcinoma.^21^ Moreover, particular stromal cell types potentially lacking CD26 expression in tumors and the molecular mechanisms underlying the decreased stromal CD26 expression have not as yet been fully elucidated.

In this study, we showed CD26 staining to be attenuated on myofibroblasts rich in tumor‐associated stroma in specimens obtained from breast cancer patients. On the cultured human breast myofibroblastic CAFs, CD26 expression is also significantly attenuated relative to that on control human mammary fibroblasts. TGF‐β and SDF‐1 autocrine signaling is responsible for the attenuated CD26 expression on these cells via Smad2/3 (Figure 4E). Given the induction of the attenuated CD26 expression on TGF‐β‐treated human mammary fibroblasts (Figure 3G‐I), different sources of TGF‐β derived from tumor cells and stromal cells^37^ in addition to CAFs may contribute to inducing downregulation of CD26 expression on mammary fibroblasts during tumor progression.

Distinct fibroblast populations with inherent functional diversity exist in stroma of human breast^38^ and skin^39^, ^40^ tissues as well as various human tumors.^41^, ^42^, ^43^ CD26 expression has been demonstrated to serve as a marker for stratification of the stromal cell type in human breast and skin tissues.^38^, ^39^, ^44^ CD105/endoglin, a coreceptor for TGF‐β family members, is also expressed on a subset of stromal myofibroblasts at the invasive borders of human colon carcinomas.^45^ Moreover, CD26^low^CD105^high^ fibroblasts with myofibroblast‐related characteristics are abundant in the terminal duct lobular unit of the human breast, while interlobular ducts are rich in CD26^high^CD105^low^ fibroblasts.^38^ Collectively, these observations indicate the importance of CD26 and CD105 expressions for identifying the particular fibroblast lineages in human mammary tissues including those of breast cancer.

We demonstrated that the attenuated CD26 staining in tumor‐associated stroma with an abundance of myofibroblasts is associated with poor outcomes for breast cancer patients, suggesting stromal CD26 staining to be a potentially novel prognostic marker. However, due to the lack of a statistically significant difference, we assume that use of another marker with stromal CD26 staining may improve prognostic power. As CD105 expression in CAFs has been indicated to mediate the activities of these fibroblasts, thereby promoting colon tumor invasion and metastasis,^45^ whether CD26^low^CD105^high^ might serve as a valuable prognostic marker must be addressed in future studies.

Increased levels of stromal TGF‐β and SDF‐1 staining are also reported to be associated with the poor outcomes in breast cancer patients.^46^, ^47^ Although these cytokines are highly produced by CAFs, as indicated earlier, it remains unclear whether CAF‐derived TGF‐β and SDF‐1 in tumors contribute to poor prognoses via downregulated stromal CD26 expression in breast cancer patients.

### 4.2 CD26 shedding on stromal fibroblasts

Shedding of membrane CD26 has been widely recognized on various cell types, such as human adipocytes, vascular smooth muscle cells (VSMCs), and peripheral blood mononuclear cells (PBMCs).^48^, ^49^ Several matrix metalloproteinases (MMPs) mediate the membrane CD26 shedding on adipocytes and VSMCs.^48^ Kallikrein‐related peptidase 5 (KLK5) also plays roles in the shedding of CD26 on human PBMCs; the CD26 shedding from PBMCs is significantly inhibited by treatment with a KLK5 inhibitor, while treatment with recombinant KLK5 has the opposite effect, dramatically inducing CD26 shedding from CD4^+^ T cells.^49^

On the other hand, DPP‐4 activity was barely detectable in media conditioned by human mammary fibroblasts and CAFs expressing a human CD26 cDNA construct. Various proteases, such as MMP2, MMP9, urokinase‐type plasminogen activator, and cathepsins potentially regulating membrane CD26 shedding are likely to be detectable in these fibroblasts according to previous reports.^50^, ^51^, ^52^, ^53^ We thus speculate that these proteases may not be responsible for CD26 shedding on human mammary fibroblasts.

### 4.3 Myofibroblastic CAFs and fibrosis‐derived myofibroblasts show inverse CD26 expression pattern

Analogies between tumors and wound tissues have been portrayed as “tumors: wounds that do not heal,” based on their similar biological processes, as exemplified by the recruitment of large numbers of myofibroblasts, extracellular matrix deposition, tissue inflammation, and angiogenesis.^54^, ^55^, ^56^ These analogous processes are also further supported by gene expression profiles between tumor‐associated stroma and tissue regeneration/repair responses.^57^, ^58^, ^59^

CD26 expression has been shown to be significantly upregulated on stromal fibroblasts associated with wounds and fibrosis.^17^, ^60^, ^61^, ^62^, ^63^ Treatment with a DPP‐4 inhibitor also attenuates the activated myofibroblastic states by inhibiting canonical TGF‐β‐Smad2/3 signaling as well as noncanonical ERK and p38 signaling.^61^, ^62^, ^64^ These findings suggest that CD26 expression is required for maintenance of the activated state on myofibroblasts present in the damaged tissues via TGF‐β signaling during regeneration and repair.

In sharp contrast, we observed significantly attenuated CD26 expression on stromal myofibroblasts in human breast carcinomas. Restoration of CD26 expression also has only a very minor influence on the TGF‐β‐Smad2/3 pathway in human breast CAFs (Figure 5C,D). Thus, this contrasting CD26 expression pattern and distinct actions modulating TGF‐β signaling serve as an exception to the well‐recognized analogies between wound‐associated fibroblasts and CAFs. To further understand the biology of CAFs, molecular insights into the mechanisms underlying the cell‐type specific roles of CD26 expression on these myofibroblasts are needed.

In summary, we obtained the unexpected findings that stromal CD26 expression is significantly attenuated through TGF‐β and SDF‐1 autocrine signaling on myofibroblastic CAFs in human breast carcinomas. As the attenuated CD26 expression in stromal myofibroblasts correlated with poor outcomes for breast cancer patients, decreased stromal CD26 expression may be useful as a prognostic marker for breast cancer patients.

## CONFLICT OF INTEREST

The authors have no potential conflict of interest to declare.
