# Supplementary figures and images for: CD26 expression is attenuated by TGF‐β and SDF‐1 autocrine signaling on stromal myofibroblasts in human breast cancers
Source: Cancer Med. 2019 May 29;8(8):3936–48. doi: 10.1002/cam4.2249 (PMC6639198; doi:10.1002/cam4.2249)

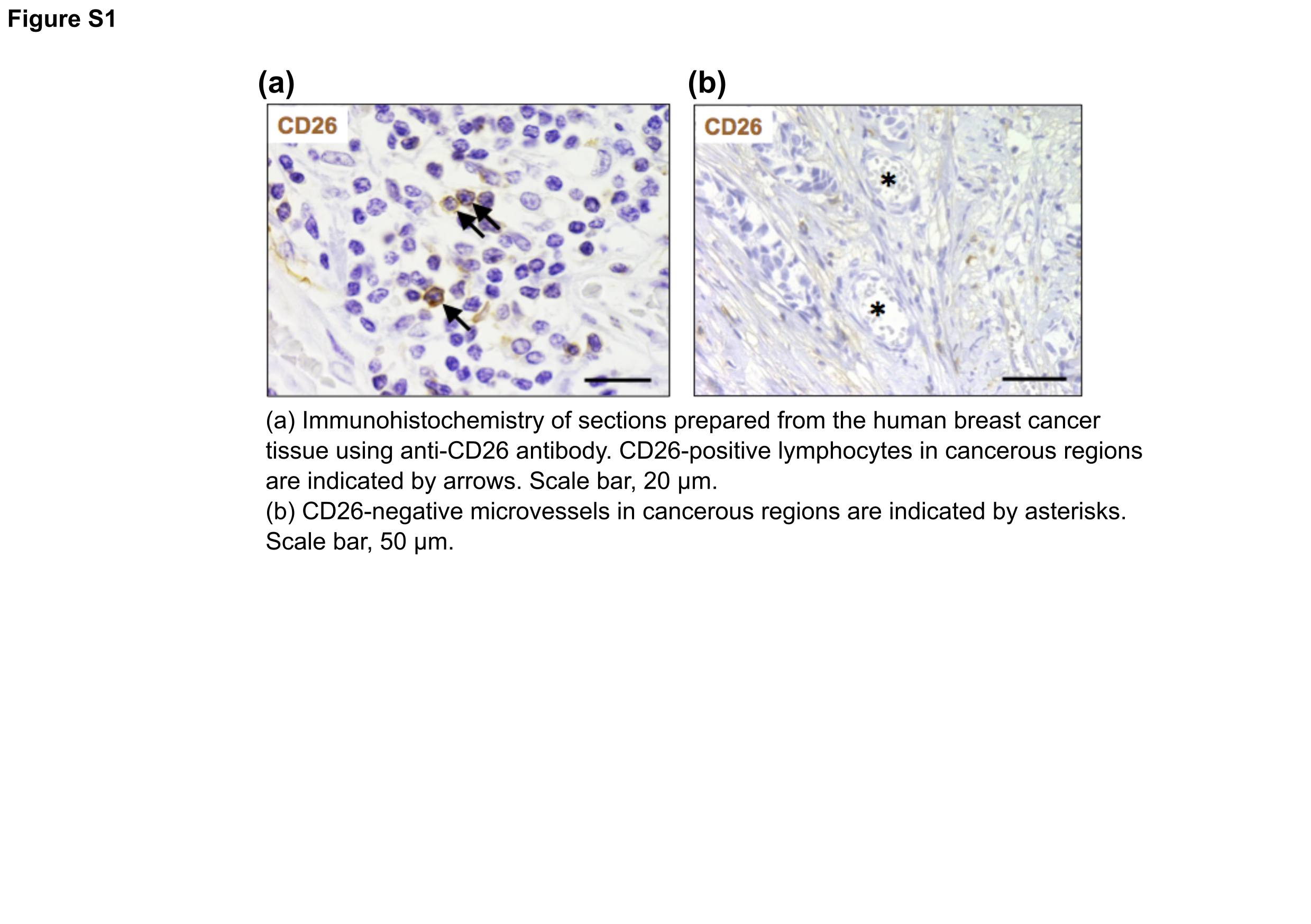

Supplement: Supplementary file 1 [file CAM4-8-3936-s001.tiff]

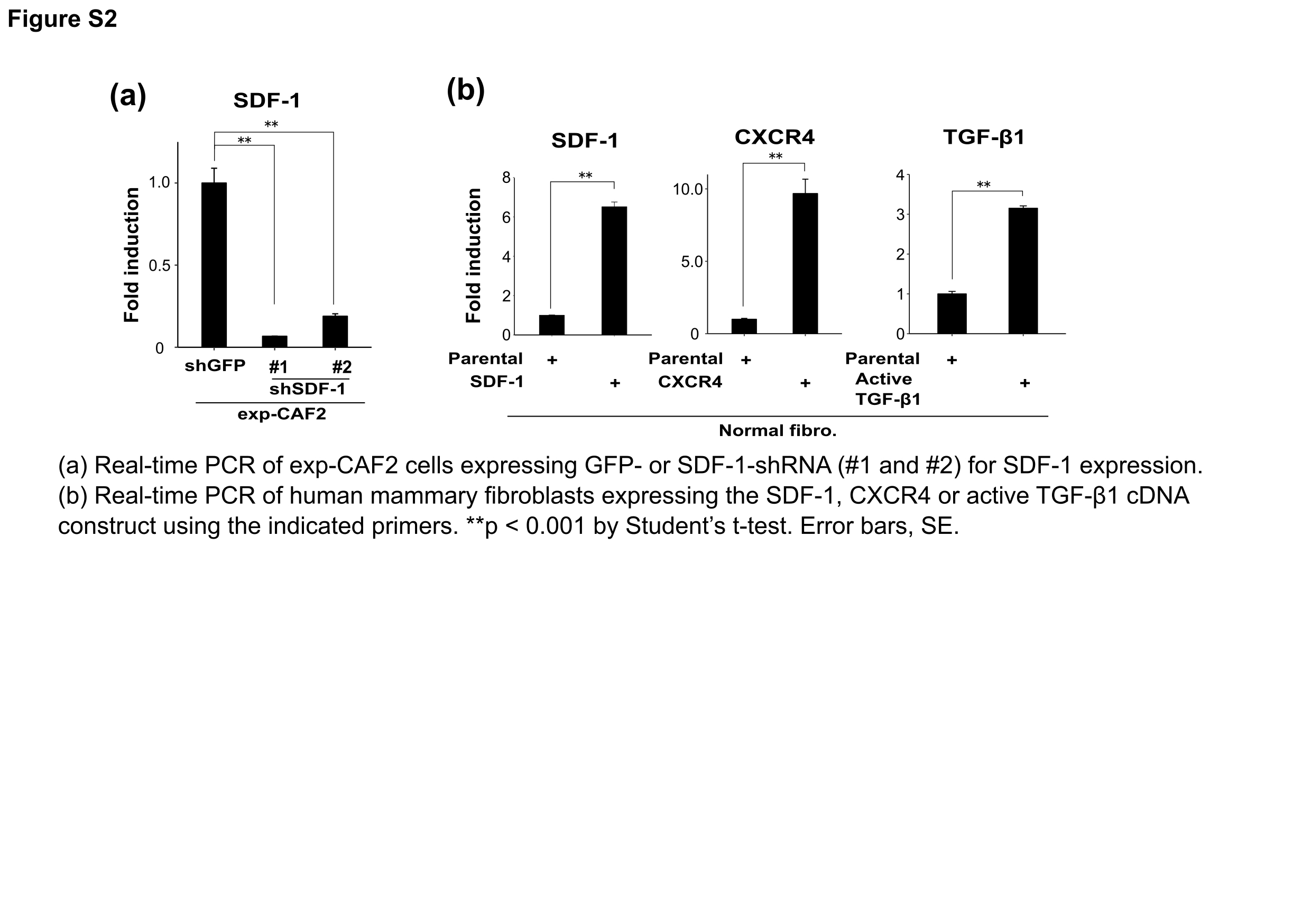

Supplement: Supplementary file 2 [file CAM4-8-3936-s002.tiff]
