# Supplementary material for: CD26 expression is attenuated by TGF‐β and SDF‐1 autocrine signaling on stromal myofibroblasts in human breast cancers
Source: Cancer Med. 2019 May 29;8(8):3936–48. doi: 10.1002/cam4.2249 (PMC6639198; doi:10.1002/cam4.2249)
Supplement: Supplementary file 3 [file CAM4-8-3936-s003.docx]

**Doc. S1 Supplementary materials and methods**

**Plasmid construction**

Human CD26 cDNA^1^ was cloned into a pBabe-neo retroviral vector. Retroviral vectors encoding human SDF-1, human CXCR4 and active swine TGF-β1 cDNA were employed as described previously.^2, 3^ tdTomato cDNA construct, kindly provided by Dr. Tsukasa Shibue (Whitehead Institute) was cloned into a pWZL-blast vector. The shRNA oligonucleotides against GFP^2^, SDF-1^2^ and Smad4^2, 4^ were generated and cloned into pLKO.1-hygro lentiviral vectors. The target sequences are described below.

**shRNA target sequences**

GFP 5’-GCAAGCTGACCCTGAAGTTCA-3’ ^2^

SDF-1 #1 5’-TCCTCGTGCTGACCGCGCTCT-3’ ^2^

SDF-1 #2 5’-AGCTATTCCTACTCTCTCCCC-3’ ^2^

Smad4 #1 5’-AGGTGTGCAGTTGGAATGTAA-3’ ^2^

Smad4 #2 5’-GCCCTATTGTTACTGTTGATG-3’ ^4^

**Retroviral and lentiviral infection**
Retroviral and lentiviral infections were performed as previously.^5, 6^ After infection, fibroblasts were selected with G418 (500 µg/ml) or hygromycin (50 µg/ml). DCIS cells and MDA-MB-231 cells were introduced pWZL-tdTomato blast vector and selected with 10 µg/ml of blasticidin. MCF10DCIS.com cells were also introduced a pBabe-human CXCR4-puro vector and selected with 2 µg/ml of puromycin.

**Real-time PCR analysis**

Total RNA was extracted using NucleoSpin RNA II (Takara) in accordance with the manufacturer’s protocol. Super Script III (Invitrogen) was used to synthesize cDNA. Real-time PCR was performed using the Fast SYBR Green Master Mix (Applied Biosystems) in triplicate in a 7500 Fast Real-Time PCR system (Applied Biosystems). Relative gene expressions were analyzed by the ΔΔCt method. Data were normalized relative to the glyceraldehyde 3-phosphate dehydrogenase gene expression level. The primer sequences are described below.

**Primer pairs for real-time PCR**
CD26 Fw 5’-AGTGGCGTGTTCAAGTGTGG-3’ ^7^

Rv 5’-CAAGGTTGTCTTCTGGAGTTGG-3’

α-SMA Fw 5’-GTGTGTGACAATGGCTCTGG-3’ ^2^
 Rv 5’-TGGTGATGATGCCATGTTCT-3’

SDF-1 Fw 5’-TGAGAGCTC GCTTTGAGTGA -3’ ^3^
 Rv 5’-CACCAGGACCTTCTGTGGAT-3’

TGF-β1 Fw 5’-ACTGCAAGTGGACATCAACG-3’ ^2^

Rv 5’-TGCGGAAGTCAATGTACAGC-3’

TGF-β2 Fw 5’- TTGACGTCTCAGCAATGGAG-3’ ^2^

Rv 5’- GGGTTCTGCAAACGAAAGAC-3’

CXCR4 Fw 5’- AATCTTCCTGCCCACCATCT-3’ ^2^

Rv 5’- GACGCCAACATAGACCACCT-3’

GAPDH Fw 5’-ACCCAGAAGACTGTGGATGG-3’ ^2^

Rv 5’-TCTAGACGGCAGGTCAGGTC-3’

**Double fluorescence immunohistochemistry**

FFPE tissue sections were prepared and subjected to antigen retrieval as described above. The slides were blocked with 5% of bovine serum albumin in PBS, and incubated with both anti-CD26 and -α-SMA antibodies or both anti-CD26 and -vimentin antibodies at 4°C overnight. After incubation with the secondary antibodies, the slides were treated with 0.2% Sudan black B dissolved in 70% ethanol for 30 min at room temperature to reduce autofluorescence. After staining with DAPI, the tissues were observed under a fluorescence Axioplan 2 microscope (Zeiss). Images were also acquired with a CCD camera and prepared using an ImageJ image analysis program.

**Western blotting analysis**

Fibroblasts were seeded at 5×10^5^ cells on a 6 cm dish and cultured in 2% FBS-containing culture medium for 24 hr prior to preparation of the whole cell lysate using SDS gel loading buffer. The lysate was separated by SDS-polyacrylamide gel electrophoresis on 12% acrylamide gel and transferred to a PVDF membrane. Primary antibodies were incubated in TBS-T (0.05 M Tris pH 7.6, 0.15 M NaCl and 0.05% Tween 20) with 10% FBS after blocking with 5% skim milk. Detection was performed using a ChemiDoc MP imaging system (BIO RAD). Signal intensity was measured using an ImageJ image analysis program.

**Flow cytometry analysis**

2×10^5^ fibroblasts were stained with anti-human CD26 mouse antibody^8^ or the isotype-matched control (MOPC-21, Sigma) in 1% FBS-PBS, followed by incubation with Alexa 647-conjugated anti-mouse IgG. After staining with 4',6-diamidino-2-phenylindole (DAPI), the specimens were subjected to flow cytometry analysis using a BD LSRFortessa cell analyzer (BD Biosciences).

**Gene expression analysis using public database**

Microarray data of primary breast CAFs and counter human mammary fibroblasts (GSE20086)^9^ were obtained from Gene Expression Omnibus. The data were subjected to Robust Multi-array Average method on R console.^10^ The averaged gene expression was calculated using non-log transformed values of gene expression in each group and shown as ratio.

**DPP-4 activity assay**

7×10^5^ human mammary fibroblasts were seeded onto a 6 cm culture dish for cell lysate preparation with lysis buffer (1% NP-40, 150 mM NaCl, 0.5 mM EDTA and 50 mM Tris pH 7.4) supplemented with protease inhibitors. For preparation of a conditioned medium, 5×10^5^ human mammary fibroblasts were cultured on a 6 cm dish for 72 hr. DPP-4 activity of the cell lysate and the conditioned medium was measured as previously described.^11^ Briefly, the specimens were incubated overnight in a 96-well plate which had been coated with anti-CD26 antibody (clone: 5F8^11^). After washing, 1 mg/ml Gly-Pro-p-nitroaniline (Sigma) was added and then the absorbance resulting from p-Nitroaniline (pNA) release was measured at 405 nm at 15 min intervals until 75 min. Serially diluted pNA (Sigma) was used for standard curve. pNA concentrations at each time points were calculated and the rate of the concentration increase was determined as DPP-4 activity (µM/min).

**Boyden chamber cell migration assay**

1.2×10^4^ DCIS cells overexpressing CXCR4 （DCIS-CXCR4） or MDA-MB-231 cells were seeded onto the upper well of a transwell chamber with an 8-µm pore size in a 24-well plate (Corning) and incubated for 10 min in the 5% CO_2_ incubator. 2.5×10^6^ fibroblasts were cultured in 6 ml of a 1:1 mixture of DMEM/F-12 GlutaMAX™ (Gibco) and DMEM high glucose GlutaMAX™ (Gibco) supplemented with 3.5% FBS and 1% PenStrep on a 10 cm dish for 24 hr. The conditioned medium was then filtered through 0.45 μm pore**-**size filters and applied to the bottoms of transwell chambers followed by incubation for 12 hr (MDA-MB-231) or 60 hr (DCIS-CXCR4). After removing the remaining cells from upper**-**side of the membrane, the transwell chambers were fixed and stained with May-Grünwald Giemsa staining solution. The number of cells that had migrated to the bottom of the membrane was counted in three independent areas in each well (n=3).

**ELISA**

exp-CAF2 cells expressing the human CD26 cDNA construct or the control empty vector were seeded at 2.5×10^5^ or 5×10^5^ cells on a 6 cm dish. The medium was replaced with 2% FBS-containing culture medium for 24 hr prior to preparation of the conditioned medium. The specimens were filtered through 0.45 μm pore**-**size filters and subjected to ELISA for SDF-1α following the manufacturer’s protocol (R&D Systems).

**Antibodies**

Antibodies (clone) Source (catalog number) Purpose

CD26 (19-32) ^8^ FCM

CD26 (5F8) ^11^ DPP-4 activity assay

CD26 R&D Systems (AF1180) WB, IHC, IF, TMA

CXCR4 (4G10) Santa Cruz Biothechnology (sc-53534) WB

α-SMA (1A4) DAKO (M0851) WB, IHC, IF, TMA

pSmad2 (138D4) Cell Signaling Technology (3108) WB

Smad2/3 (18/Smad2/3) BD Bioscience (610843) WB

Smad4 (B-8) Santa Cruz Biothechnology (sc-7966) WB

α-tubulin (B-5-1-2) Sigma (T5168) WB

Vimentin (V9) DAKO (IS630) IF
EnVision+ System-HRP Labelled Polymer Anti-mouse

DAKO (K4001) WB, IHC

EnVision+ System-HRP Labelled Polymer Anti-rabbit

DAKO (K4003) WB

Donkey anti-Goat IgG-A488 Thermo Fisher Scientific (A-11055) IF
Donkey anti-Mouse IgG-A594 Thermo Fisher Scientific (A-21203) IF

Goat anti-Mouse IgG-A647 Thermo Fisher Scientific (A-21236) FCM

Polyclonal rabbit anti-goat immunoglobulins/HRP DAKO (P0163) WB

Histo-fine simple stain MAX-PO(G) Nichirei bioscience (H1403) IHC

**Chemical reagents**

Recombinant human TGF-β1 R&D Systems (240-B-010)

SB431542 Sigma (S4317)

Gly-Pro p-nitroanilide p-toluenesulfonate salt Sigma (G2901)

p-Nitroaniline Sigma (185310)

**References**

[1] Tanaka T, Camerini D, Seed B, et al. Cloning and functional expression of the T cell activation antigen CD26. J Immunol. 1992; 149: 481-6.

[2] Kojima Y, Acar A, Eaton EN, et al. Autocrine TGF-beta and stromal cell-derived factor-1 (SDF-1) signaling drives the evolution of tumor-promoting mammary stromal myofibroblasts. Proc Natl Acad Sci U S A. 2010; 107: 20009-14.

[3] Orimo A, Gupta PB, Sgroi DC, et al. Stromal fibroblasts present in invasive human breast carcinomas promote tumor growth and angiogenesis through elevated SDF-1/CXCL12 secretion. Cell. 2005; 121: 335-48.

[4] Koinuma D, Tsutsumi S, Kamimura N, Imamura T, Aburatani H, Miyazono K. Promoter-wide analysis of Smad4 binding sites in human epithelial cells. Cancer Sci. 2009; 100: 2133-42.

[5] Elenbaas B, Spirio L, Koerner F, et al. Human breast cancer cells generated by oncogenic transformation of primary mammary epithelial cells. Genes Dev. 2001; 15: 50-65.

[6] Stewart SA, Dykxhoorn DM, Palliser D, et al. Lentivirus-delivered stable gene silencing by RNAi in primary cells. RNA. 2003; 9: 493-501.

[7] Varona A, Blanco L, Perez I, et al. Expression and activity profiles of DPP IV/CD26 and NEP/CD10 glycoproteins in the human renal cancer are tumor-type dependent. BMC Cancer. 2010; 10: 193.

[8] Hatano R, Yamada T, Matsuoka S, et al. Establishment of monoclonal anti-human CD26 antibodies suitable for immunostaining of formalin-fixed tissue. Diagn Pathol. 2014; 9: 30.

[9] Bauer M, Su G, Casper C, He R, Rehrauer W, Friedl A. Heterogeneity of gene expression in stromal fibroblasts of human breast carcinomas and normal breast. Oncogene. 2010; 29: 1732-40.

[10] Irizarry RA, Hobbs B, Collin F, et al. Exploration, normalization, and summaries of high density oligonucleotide array probe level data. Biostatistics. 2003; 4: 249-64.

[11] Hosono O, Homma T, Kobayashi H, et al. Decreased dipeptidyl peptidase IV enzyme activity of plasma soluble CD26 and its inverse correlation with HIV-1 RNA in HIV-1 infected individuals. Clin Immunol. 1999; 91: 283-95.
